# Sepsis surveillance: an examination of parameter sensitivity and alert reliability

**DOI:** 10.1093/jamiaopen/ooz014

**Published:** 2019-06-11

**Authors:** Robert C Amland, Mark Burghart, J Marc Overhage

**Affiliations:** Population Health, Cerner Corporation, Kansas City, Missouri, USA

**Keywords:** sepsis, classification, expert systems, decision support, ergonomics

## Abstract

**Objective:**

To examine performance of a sepsis surveillance system in a simulated environment where modifications to parameters and settings for identification of at-risk patients can be explored in-depth.

**Materials and Methods:**

This was a multiple center observational cohort study. The study population comprised 14 917 adults hospitalized in 2016. An expert-driven rules algorithm was applied against 15.1 million data points to simulate a system with binary notification of sepsis events. Three system scenarios were examined: a scenario as derived from the second version of the Consensus Definitions for Sepsis and Septic Shock (SEP-2), the same scenario but without systolic blood pressure (SBP) decrease criteria (near SEP-2), and a conservative scenario with limited parameters. Patients identified by scenarios as being at-risk for sepsis were assessed for suspected infection. Multivariate binary logistic regression models estimated mortality risk among patients with suspected infection.

**Results:**

First, the SEP-2-based scenario had a hyperactive, unreliable parameter SBP decrease >40 mm Hg from baseline. Second, the near SEP-2 scenario demonstrated adequate reliability and sensitivity. Third, the conservative scenario had modestly higher reliability, but sensitivity degraded quickly. Parameters differed in predicting mortality risk and represented a substitution effect between scenarios.

**Discussion:**

Configuration of parameters and alert criteria have implications for patient identification and predicted outcomes.

**Conclusion:**

Performance of scenarios was associated with scenario design. A single hyperactive, unreliable parameter may negatively influence adoption of the system. A trade-off between modest improvements in alert reliability corresponded to a steep decline in condition sensitivity in scenarios explored.

KEY FINDINGS

- Omission error can be minimized by establishing an adequate reliability metric for adoption, with an understanding that performance of parameters is not homogenous.
- Prior to modifying system parameter configurations, realize that small improvements in reliability may be offset by a corresponding steep decline in condition sensitivity.
- Constraining systems by design promotes substitution effects on parameters that increase mortality risk for a sizable subgroup of patients.

## BACKGROUND AND SIGNIFICANCE

Despite increased awareness of sepsis, accurately identifying patients at risk remains a challenge.^1^ This phenomenon is applicable to sepsis surveillance systems, noteworthy for having characteristic heterogenous designs. However, commonalities across systems typically include a limited number of parameters and alert criteria threshold settings selected to attain high reliability at a detrimental cost of declining sensitivity.^2–4^ A countervailing approach is a broadening of parameter inclusion applicable to sepsis surveillance, which could play a larger role in promoting early recognition and treatment, and improving the likelihood of survival.^5^

A sepsis surveillance system is typically available in real-time at the point of care and integrates clinical decision support functionality into the clinical workflow.^6^ The system continuously screens a patient’s physiological data and is capable of delivering a notification to a provider within minutes of activation.^7–9^ Screening typically begins when the patient presents to the emergency department or is otherwise admitted to the hospital, and runs uninterrupted until discharge.^10^^,^^11^

Although many surveillance systems have good performance characteristics, none are 100% accurate. The system’s usefulness is premised upon a provider’s subjective perception of reliability, which in itself is associated with the design of system parameters and alert criteria thresholds.^12^ A reliable alert reduces harmful cry-wolf effects, such as ignoring alert notifications or delays in response.^13^ Moreover, a surveillance system characterized by an adequate alert reliability (eg, ≥70% positive predictive value [PPV]) increases the likelihood of response to alert notifications and attaining protocol compliance.^14^ In contrast, providers’ reliance on the system to accurately screen-in at-risk patients is supported by systems possessing high condition sensitivity. Nevertheless, an intentionally sensitive high-reliance design may render the system prone to false positives and an unacceptably low reliability metric, a trade-off which has implications for adoption and use.^15–17^

Two types of error tensions are inherent in surveillance systems. First, commission error (ie, over-diagnosing or treatment despite the absence of the condition; false positive) is observable when an alert activates and delivers a notification to a provider who responds despite the alert being in error. Second, an omission error (ie, missed detection and nonresponse; false negative) occurs when a patient is condition positive, but the system remains silent and a provider does not place an order. Designers of surveillance systems, therefore, may rely upon evidence and heuristics to increase reliability to reduce commission errors while hedging condition sensitivity given an uncertain omission error potential.^18^

## OBJECTIVES

The objective of this study was to examine performance of a sepsis surveillance system in a simulated environment, where modifications to parameters and alert criteria thresholds to accurately screen-in at-risk patients could be explored. Although early guidance suggested surveillance system performance can be improved substantially by manipulating parameters and alert criteria thresholds, a small adjustment may have an unintentional, detrimental impact on condition sensitivity and become an exercise in frustration.^19^ Moreover, the impact of excluding parameters on performance when compared to a system as derived from the second version of the Consensus Definitions for Sepsis and Septic Shock (SEP-2) is generally unknown.^20^

## MATERIALS AND METHODS

### Study design

This was a retrospective multiple center observational cohort study. The study site was eight hospitals located in two different regions in southwest USA. All facilities had an enterprise electronic health record (EHR) system (Millennium: Cerner Corporation, Kansas City, MO, USA). This study was approved with waiver of informed consent by the Western Institutional Review Board.

The study population included 14 917 patients (≥18 years) admitted to the hospital and discharged during a 90-day observation period in 2016. Data were retrieved from an enterprise data warehouse and production system. To account for updates to physiological data, a counter-like key written in SQL was instituted during cleaning and processing of data. The counter-like key was used to sort multiple instances of updated physiological data related to a clinical event. Essentially, this data engineering process allowed for a proper temporal sequencing of many instances related to a single clinical event and important to classification of physiological data. To merge data extracts, a common unique encounter identifier created by the EHR system was available as the primary key.

**Figure 1.**
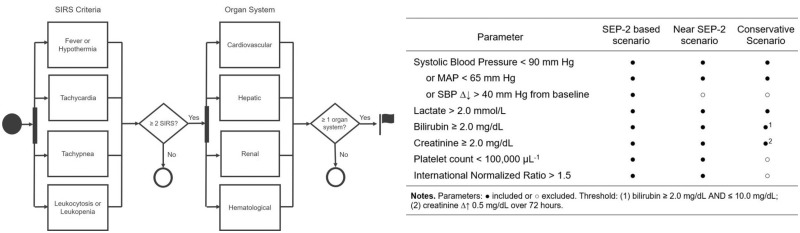
Sepsis surveillance model and corresponding organ system parameters by surveillance system scenario. SBP: systolic blood pressure; SEP-2: Consensus Definitions for Sepsis and Septic Shock; SIRS: Systemic Inflammatory Response Syndrome; MAP: mean arterial pressure.

All patient encounters were examined for suspicion of infection, which we defined as microbiology cultures as having been drawn and intravenous (IV) antibiotics administered.^21^^,^^22^ To identify patients at-risk for sepsis, an expert-driven rules algorithm was developed to simulate a sepsis surveillance system with a binary alert notification. Figure 1 illustrates the foundation model with three scenarios for classification. The SEP-2-based scenario of the model defined the universe of physiological data available for classification, which comprised 15.1 million data points.

The SEP-2-based scenario adhered closely to the definition of severe sepsis recognized by the Centers for Medicare & Medicaid Services.^23^ Each scenario thereafter introduced modifications to parameters and/or adjustments to alert criteria thresholds to potentially improve the trade-off between alert reliability and condition sensitivity. The near SEP-2 scenario excluded the parameter corresponding to a decline in systolic blood pressure (SBP) >40 mm Hg from the SEP-2-based scenario because some patients may have experienced adrenergic stress-induced elevation in blood pressure, and if normalized, could erroneously activate a severe sepsis alert.^24^ In addition to excluding the parameter SBP decrease >40 mm Hg, the conservative scenario modified four parameters: an increase of 0.5 mg/dL over 72 h for creatinine; excluded bilirubin >10 mg/dL; and eliminated platelets and international normalized ratio (INR) because these parameters may also indicate primary liver injury.^25–27^

#### Definitions

The primary outcome was identification of patients with severe sepsis, defined as suspected or confirmed infection with clinical evidence of two Systemic Inflammatory Response Syndrome (SIRS) criteria and at-least one organ system dysfunction. A secondary outcome, mortality, was defined as in-hospital death or referral to hospice at discharge.

As illustrated in Figure 1, SIRS was indicated when two of the following four criteria were satisfied: (1) temperature >38.3°C or <36°C; (2) heart rate >90 beats/min; (3) respiratory rate >20 breaths/min; and (4) white blood cell count >12 000 cells/mm^3^, <4000 cells/mm^3^, or >10% immature (band) forms. Severe sepsis screen-in was established when ≥2 SIRS criteria were present, and ≥1 of the following four organ system dysfunction criteria were satisfied: (1) SBP <90 mm Hg, or mean arterial pressure <65 mm Hg, or SBP decrease >40 mm Hg from baseline, or serum lactate >2.0 mmol/L; (2) total bilirubin: ≥2.0 mg/dL; (3) serum creatinine: ≥2.0 mg/dL; and (4) platelet count <100 000 µL^−1^ or INR >1.5. Organ dysfunction was indicated by itemized parameter criteria by system scenario shown in Figure 1.

Classification of parameters for each system scenario applied the following lookback periods: 12 h for lactate, 72 h for creatinine, and 30 h for the remaining criteria. The first SBP reading within the 30-h lookback period was established as the baseline SBP for the parameter SBP decrease >40 mm Hg from baseline.

Suspected infection was defined by the combination of microbiology cultures drawn and anti-infective antibiotics administered. Microbiology cultures included blood, body fluid, bronchial, catheter tip, cerebrospinal fluid, fungal, ova and parasites, sputum, stool, tissue, urine, and wound. Antibiotics included ampicillin-sulbactam, azithromycin, cefepime, ceftriaxone, ciprofloxacin, clindamycin, fluconazole, fluticasone-salmeterol, levofloxacin, meropenem, piperacillin-tazobactam, and vancomycin.

Demographics and severity of illness measures were calculated at the time of patient arrival to the hospital. The National Early Warning (NEWS) composite acuity score was calculated and categorized by using the first vital signs and neurologic assessment documented, to include respiratory rate, oxygen saturation, temperature, SBP, heart rate, and level of consciousness.^28^^,^^29^ An indication of severe electrolyte abnormality and metabolic disturbance was calculated using the *apparent* Strong Ion Difference (SIDa), where corrected SIDa = [(Na^+^ + K^+^ + 1.85) − Cl^−^]; and flagged when SIDa ≤34.0 or ≥48.0 mmol/L.^30–33^

### Statistical analysis

Data were retrospectively analyzed. A confusion matrix, noteworthy for its specific 2 by 2 table layout, was created to calculate performance metrics (ie, PPV and sensitivity) for each system scenario on suspected infection. Multivariate binary logistic regression (MLR) modeling was used to estimate mortality risk among patients with suspected infection. Adjusted odds ratios with 95% confidence intervals were reported for each parameter by system scenario and illustrated in a forest plot. For the parameter included in each MLR model, the absence of that parameter was the reference value. MLR model covariates included patient age, sex, and NEWS acuity category. Classification of parameters was coded in Python (version 3.6) using Scikit-learn (version 0.19.2) and Pandas (version 21.1) libraries. The analysis of statistical data was conducted in SPSS v25 (IBM, Inc., Armonk, NY, USA).

## RESULTS

Classification output merged into the study population analytic set identified 3862 patients who met criteria associated with the SEP-2-based scenario. Characteristics of these patients are shown in Table 1. Less than two-thirds (*n *=* *2472 of 3862; 64%) of patients had a suspicion of infection, with lactate measured among 81% (*n *=* *1999 of 2472) of patients. In comparison, a relative 19% [(3142–3862)/3862] fewer patients and relative 33% [(2598–3862)/3862] fewer patients were identified by the near SEP-2 scenario or the conservative scenario, respectively. Performance of system scenarios showed alert reliability of 64%, 68%, and 71% PPV on suspected infection, while sensitivity was 100%, 87%, and 75% for the SEP-2-based scenario, the near SEP-2 scenario, and the conservative scenario, respectively.

**Table 1.** Characteristics of patients with severe sepsis flag by system scenario

Characteristics	SEP-2-based scenario	Near SEP-2 scenario	Conservative scenario
	*N* (%)	*N* (%)	*N* (%)
Hospitalizations	3862 (100)	3142 (100)	2598 (100)
Suspected infection	2472 (64)	2147 (68)	1843 (71)
Lactates measured	1999 (81)	1797 (84)	1591 (86)
Demographics			
Age (y), median (IQR)	66 (52–76)	66 (53–77)	65 (52–76)
Female gender	1173 (48)	995 (46)	854 (46)
First clinical results			
SIDa ≤34 or ≥48 mmol/L	128 (05)	125 (06)	105 (06)
NEWS composite score			
0–4 points	1469 (59)	1243 (58)	1016 (55)
5–6	465 (19)	418 (19)	371 (20)
7–8	301 (12)	260 (12)	242 (13)
9–25	237 (10)	226 (11)	214 (12)
Clinical outcomes			
Expired or hospice	424 (17)	404 (19)	380 (21)
ICU, expired, hospice	848 (34)	787 (37)	734 (40)
LOS (d), median (IQR)	4 (3–9)	5 (3–9)	5 (3–9)

*Note:* Suspected infection elements include microbiology cultures drawn and anti-infective intravenous antibiotics given. ICU admission within 48 h after arrival.

*Abbreviations:* ICU: intensive care unit; IQR: interquartile range; LOS: length of stay; NEWS: National Early Warning Score; SEP-2: Consensus Definitions for Sepsis and Septic Shock; SIDa: *apparent* Strong Ion Difference.

The typical patient with suspected infection was 66 years old and slightly less likely to be female. Upon arrival to the hospital, approximately 5% patients had severe electrolyte abnormality and metabolic disturbance; and one in four patients had NEWS ≥7 points of which half of them had NEWS ≥9 points, which suggested a progressive physiologic deterioration. Between 17% and 21% patients either expired in-hospital or referred to hospice at discharge.

Table 2 describes the occurrence of patients by classification and proportion with suspected infection, which ranged from 50% to 85%. The parameter SBP decrease >40 mm Hg from baseline was responsible for identifying the greatest number of patients in the SEP-2-based scenario and demonstrated low reliability on suspected infection and therefore drove a large proportion of the false positive alerts. Classification of parameters on the other two scenarios showed not only a notable relative decline in detected patients as the scenarios became increasingly constrained, but also a complex substitution effect on parameters between scenarios.

**Table 2.** Patients with severe sepsis flag and suspicion of infection

	SEP-2-based scenario		Near SEP-2 scenario		Conservative scenario	
Parameter	*N*	*n* (%)	*N*	*n* (%)	*N*	*n* (%)
SBP Δ↓	1083	577 (53)	–		–	
SBP or MAP	719	435 (61)	771	447 (58)	1185	745 (63)
Lactate	759	643 (85)	712	596 (84)	861	729 (85)
Bilirubin	121	69 (57)	120	66 (55)	222	136 (61)
Creatinine	88	44 (50)	357	258 (72)	174	105 (60)
MODS	394	283 (72)	522	383 (73)	156	128 (82)
Platelets	278	185 (67)	255	168 (66)	–	
INR	420	236 (56)	405	229 (57)	–	

*Note:* Parameter SBP Δ↓: SBP decrease >40 mm Hg from baseline.

*Abbreviations:* INR: international normalized ratio; MAP: mean arterial pressure; MODS: multiple organ dysfunction; SBP: systolic blood pressure; SEP-2: Consensus Definitions for Sepsis and Septic Shock.

In the SEP-2-based scenario, 66% (*n *=* *2561 of 3862) patients had a cardiovascular system parameter indicated compared to 47% (*n *=* *1483 of 3142) patients in the near SEP-2 scenario and 77% (*n *=* *2046 of 2598) patients in the conservative scenario. Moreover, 5% (*n *=* *209) patients had a hepatic or renal system parameter indicated in the SEP-2-based scenario compared to 15% (*n *=* *477) and 15% (*n *=* *396) patients in the near SEP-2 scenario and conservative scenario, respectively. Finally, 10% (*n *=* *394) patients had multiple organ dysfunction (MODS) indicated in the SEP-2-based scenario compared to 17% (*n *=* *522) patients in the near SEP-2 scenario and 6% (*n *=* *156) patients in the conservative scenario.

### Substitution effects on parameters among patients with suspected infection

#### Membership in both SEP-2-based scenario and near SEP-2 scenario

Approximately 87% patients identified by the SEP-2-based scenario who had a suspicion of infection were also identified by the near SEP-2 scenario. Among these patients, one in four had a different classification in the near SEP-2 scenario than their initial classification in the SEP-2-based scenario (*n *=* *540 of 2147; 25%). Of the 540-patient subgroup with a change in classification, the new parameter included 27% cardiovascular, 1% hepatic, 40% renal, 5% hematological, and 27% MODS. The crude mortality rate was 22% (*n *=* *118 of 540) versus 18% (*n *=* *286 of 1607) in the 540-patient subgroup compared to the other patient subgroup, respectively. When compared with patients who had the same classification between system scenarios, patients with a new classification in the near SEP-2 scenario had 29% increased mortality risk (unadjusted odds ratio 1.29, 95% confidence interval 1.02–1.64).

#### Membership in both SEP-2-based scenario and conservative system

Approximately 75% patients identified by the SEP-2-based scenario who had a suspicion of infection were also identified by the conservative scenario. Among these dual membership patients, one in three had a different classification in the conservative scenario than their initial classification in the SEP-2-based scenario (*n *=* *646 of 1843; 35%). Of the 646-patient subgroup with a change in classification, the new parameter included 74% cardiovascular, 11% hepatic, 10% renal, and 5% MODS. The crude mortality rate was 26% (*n *=* *165 of 646) versus 18% (*n *=* *215 of 1197) in the 646-patient subgroup compared to the other patient subgroup. When compared with patients who had the same classification between scenarios, patients with a new classification in the conservative scenario had 57% increased mortality risk (unadjusted odds ratio 1.57, 95% confidence interval 1.24–1.97).

#### Mortality outcomes

**Figure 2.**
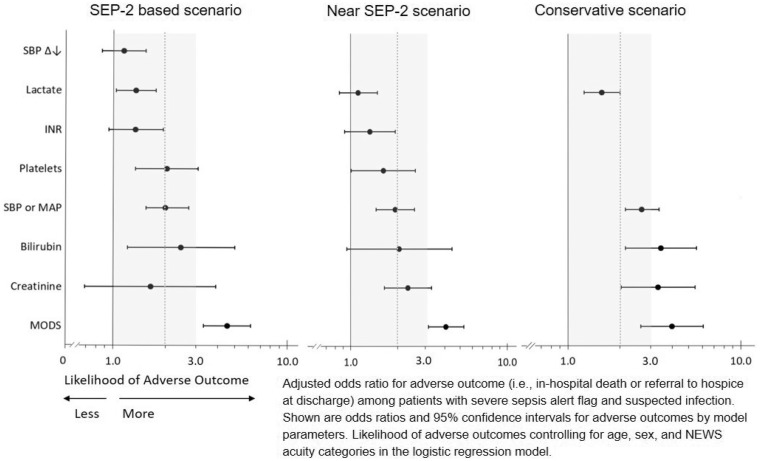
Forest plot of organ system parameters on mortality by surveillance system scenario. INR: international normalized ratio; MAP: mean arterial pressure; MODS: multiple organ dysfunction; NEWS: National Early Warning Score; SBP: systolic blood pressure; SEP-2: Consensus Definitions for Sepsis and Septic Shock.

In multivariable analysis, risk-adjusted mortality outcomes among patients with suspected infection differed by classification of parameter. Forest plots illustrated in Figure 2 illuminate mortality risk by parameter within respective system scenarios. An increase in mortality risk was pronounced by the parameter creatinine when comparing the near SEP-2 scenario to the SEP-2-based scenario. Moreover, mortality risk increased for each parameter, with exception of the parameter MODS, when comparing the conservative scenario to the SEP-2-based scenario.

## DISCUSSION

Finding an agreeable trade-off between reliability and sensitivity in a sepsis surveillance system can be challenging, especially when the objective is to achieve a target performance metric of 70% PPV on suspected infection. The simulation illustrated the SEP-2-based scenario experienced performance issues pertinent to a hyperactive, unreliable parameter SBP decrease >40 mm Hg from baseline. By excluding this parameter, a corresponding substitution effect increased alert reliability modestly but lowered sensitivity more dramatically. Mortality risk increased significantly as system configurations became more conservative. This said, the near SEP-2 scenario achieved an adequate trade-off between alert reliability and condition sensitivity particularly when considering mortality risk.

This study is potentially the first study to simulate a sepsis surveillance system that applied scenarios to gain insights into alert activation, performance, and mortality risk. In this regard, the study’s findings offer a unique contribution to the literature. The study, nevertheless, has limitations to consider. This was a multiple center observational cohort study involving eight hospitals in the USA. A classification of parameters using structured clinical data occurred 6–9 months after launch of the hospitals’ sepsis management programs, which may have introduced informed presence or other selection bias associated with real-world clinical practice.^34^ The accuracy and timeliness of clinical documentation at the patient bedside were not examined; however, multiple instances of clinical results for a same order were identified retrospectively while cleaning and processing of physiological data. Therefore, a counter-like key was coded and instituted when processing data to establish the proper temporal sequence of updated clinical results. Simulation results may not be applicable to other hospitals or health systems.

Sepsis surveillance is an effective approach for reducing mortality risk among at-risk patients.^14^^,^^35^^,^^36^ This study’s findings support the enduring robustness of severe sepsis definition articulated in policy,^23^ although room for improving precision exists. Despite being on solid ground when referencing a well-established sepsis surveillance model, a system’s clinical effectiveness may be questioned when a hyperactive unreliable parameter exists on the one-hand, or a more conservatively parameterized design prevails. One aberrant parameter may be enough to produce a negative perception of the whole system. In contrast, a conservatively parameterized system can increase reliability and garner positive perceptions of effectiveness in the near-term, but good-willed perceptions over-time may result in incremental changes that degrade performance.^9^ In this regard, condition sensitivity can be improved at a cost of reducing alert reliability in a conservative system, with full-realization that options to improve performance are already limited before creating negative perceptions.

In this light, a SEP-2-based scenario was the intended target system. But, study findings showed a hyperactive parameter SBP decrease >40 mm Hg from baseline and had inadequate reliability on suspected infection. The issue pertained to the difficulty in defining a rule for accurate classification of hypotension, considering the existence of substantial variability in time intervals for SBP readings coupled with a myriad of possibilities for an SBP decrease >40 mm Hg. This said, the system’s reliability improved to an adequate level for response after excluding that parameter. Further examination on the intersection of management of high blood pressure and SIRS is necessary before reintroducing the parameter SBP decrease >40 mm Hg from baseline into a sepsis surveillance system.

Several machine learned models to identify patients at-risk for sepsis in the intensive care unit have been published.^37–39^ Models have been developed using a variety of definitions of severe sepsis,^20^^,^^40^ and demonstrated a similar sensitivity (ie, 80–85%) to the near SEP-2 scenario but a lower alert reliability (ie, 20–30% PPV). The near SEP-2 scenario coupled with a clinical suspicion of infection may be a reliable outcome measure to use for machine learning training and testing.^41^

Future research might include model development using the near SEP-2 scenario with suspected infection as a primary target as well as secondary targets defined by the seven organ dysfunction parameters. Given surveillance systems are not 100% accurate, investigators should examine system performance driven by a machine learning-based algorithm coupled with a backstop expert-driven rules algorithm.

## CONCLUSION

Performance of the sepsis surveillance system was associated with its design. A substitution effect on parameters occurred among a subgroup of patients as systems became more conservative. Mortality risk increased significantly when systems applied constraints. This phenomenon suggests the likelihood of survival improves when a sepsis surveillance system has sufficiently adequate reliability that minimizes omission error.

## DATA AVAILABILITY

Data available from the Dryad Digital Repository: https://dx.doi.org/10.5061/dryad.vh380vb.

## CONTRIBUTORS

All authors contributed to the study design, analysis, or interpretation of data; R.C.A. and M.B. were responsible for data acquisition. All authors participated in drafting the manuscript for important intellectual content. All authors approved the final version of the manuscript.
